# Antimalarial Properties of Aqueous Crude Extracts of* Gynostemma pentaphyllum* and* Moringa oleifera* Leaves in Combination with Artesunate in* Plasmodium berghei*-Infected Mice

**DOI:** 10.1155/2016/8031392

**Published:** 2016-10-31

**Authors:** Voravuth Somsak, Preeyanuch Borkaew, Chokdee Klubsri, Kittiyaporn Dondee, Panatda Bootprom, Butsarat Saiphet

**Affiliations:** Department of Clinical Chemistry, Faculty of Medical Technology, Western University, Kanchanaburi 71170, Thailand

## Abstract

Due to the emergence and spread of malaria parasite with resistance to antimalarial drugs, discovery and development of new, safe, and affordable antimalarial are urgently needed. In this respect, medicinal plant extracts are targets to optimize antimalarial actions and restore efficacy of standard antimalarial drugs. The present study was aimed at determining the antimalarial activities of* Gynostemma pentaphyllum* and* Moringa oleifera* leaf extracts in combination with artesunate against* Plasmodium berghei*-infected mice.* P. berghei* ANKA maintained by serial passage in ICR mice were used based on intraperitoneal injection of 1 × 10^7^ parasitized erythrocytes and subsequent development of parasitemia. These infected mice were used to investigate the antimalarial activity of artesunate (6 mg/kg) in combination with 500, 1,000, and 2,000 mg/kg of* G. pentaphyllum* and* M. oleifera* leaf extracts using 4-day suppressive test. It was found that these extracts showed significant (*P* < 0.05) antimalarial activity in dose-dependent manner with percentage of suppression of 45, 50, and 55% for* G. pentaphyllum* leaf extract and 35, 40, and 50% for* M. oleifera* leaf extract. Additionally, artesunate combined with these extracts presented higher antimalarial activity, compared to extract treated alone with percentage of suppression of 78, 91, and 96% for* G. pentaphyllum* leaf extract and 73, 82, and 91% for* M. oleifera* leaf extract. The results indicated that combination treatment of* G. pentaphyllum* or* M. oleifera* leaf extracts with artesunate was able to increase the antimalarial activity by using low dose of artesunate. Hence, these results justified the combination of these extracts and artesunate in antimalarial herbal remedies.

## 1. Introduction

Malaria is a major parasitic disease with high mortality and morbidity, especially in the sub-Saharan Africa, Latin America, and Asia. About 3 billion people worldwide are exposed annually, with 1.2 billion at high risk, and some 200 million developed symptomatic malaria. Moreover, it was estimated that 1 million deaths have occurred in the world [1]. This disease is caused by malarial protozoa parasite from genus* Plasmodium* and transmitted by female* Anopheles* mosquito. Due to the lack of effective vaccine to prevent malaria, the global strategy for malaria mainly focuses on case management through provision of antimalarial drugs which are capable of reducing or eliminating malaria parasites. Unfortunately, malaria parasite has developed resistance to drugs used in malarial therapy except the artemisinin [2]. However, artemisinin produces fast recrudescence when used alone due to its short half-life. Hence, artemisinin used in combination with other antimalarials, a combination known as artemisinin-combination therapy (ACT), has been recommended [3]. In addition, resistance of mosquitoes to insecticides has led to an increase in severe malaria, complicating the eradication of the disease, and the resurgence of malaria [4]. Moreover, many antimalarials in use today have high toxicity that exposes patients' health expenditure [5]. In this respect, medicinal plant extracts are potential targets [6]. Although up to 80% of Thailand population uses traditional medicinal plants for management of diseases, these plants are not yet fully explored [7]. Various researches have been conducted to investigate the antimalarial efficacy of traditionally used plants in Asia and Africa. For example, Ramazani et al. worked on ten Iranian plant species but only* Boerhavia elegans*,* Solanum surattense*, and* Prosopis juliflora* showed a promising antimalarial activity* in vitro* and* in vivo* with no toxicity [8]. Verma et al. reported that* Holarrhena antidysenterica* and* Viola canescens* exhibited* in vitro* antimalarial activity [9].

*Gynostemma pentaphyllum* and* Moringa oleifera* have been used traditionally for treatment of several diseases involving oxidative stress and infection [10, 11]. Many reports have described the activities of these plant extracts, including antioxidant, anti-inflammation, anticancer, antiparasitic, antidiabetes, antibacterial activities, and homeostasis of biomolecules in blood [12–16]. Recently, these plant extracts were reported to exert antimalarial activity in mouse model [17]. However, combination therapy with standard antimalarial drug, artesunate, has not yet been studied using these plant extracts. Therefore, the aim of the present study is to investigate antimalarial activities of* G. pentaphyllum* and* M. oleifera* extracts in combination with artesunate in* P. berghei*-infected mice.

## 2. Materials and Methods

### 2.1. Artesunate

The pure artesunate powder used in this study was a gift from Dr. Chairat Uthaipibull, National Center for Genetic Engineering and Biotechnology (BIOTEC). It was dissolved in 7% Tween 80 and 3% ethanol in distilled water to provide dose of 6 mg/kg. This dose was based on the ED50 of artesunate on* P. berghei*-infected mice [18].

### 2.2. Plant Materials

Dried leaves of* G. pentaphyllum* and* M. oleifera* were purchased from the Royal Project Foundation shop at Suphan Buri province. The plant materials were identified by Dr. Sakaewan Ounjaijean at Faculty of Pharmacology, Payap University, and voucher specimens were deposited in Department of Clinical Chemistry, Faculty of Medical Technology, Western University, with voucher numbers CC-MT-WTU01 (*G. pentaphyllum*) and CC-MT-WTU02 (*M. oleifera*).

### 2.3. Preparation of Aqueous Crude Extracts

For preparation of crude extract, microwave-associated hot water extract was carried out as previously described [19]. Briefly, dried leaves of plant materials were ground using electric blender to obtain the powdered dried plant materials and subsequently dissolved in distilled water at the proportion of 1 : 10. The mixtures were heated in microwave at 360 W for 5 min and incubated at room temperature for overnight to obtain complete extraction. Filter through Whatman no. 1 filter paper was done, and filtrate was then freeze-dried to obtain aqueous crude extracts of* G. pentaphyllum* (GPE) and* M. oleifera* (MOE). The extracts were stored at −20°C until used. Before use, the extracts were dissolved in a mixture of 7% Tween 80 and 3% ethanol in distilled water to provide appropriate doses.

### 2.4. Experimental Mice

Pathogen-free female ICR mice, 4 weeks old, weighting 20–25 g, obtained from the National Laboratory Animal Center, Mahidol University, Thailand, were used. They were kept at the animal room with temperature within 25–28°C and 12 h light/12 h dark cycle. They were fed with standard diet pellet CP082 and drinking water* ad libitum*. All experiments involving animals were approved and ratified by the Animal Ethical Committee, Western University.

### 2.5. Acute Toxicity Test

GPE and MOE were evaluated for their acute toxicity in experimental mice as previously described with some modification [20]. Briefly, the mice were starved for 3-4 h before the experiment with only drinking water allowed and 1-2 h after the administration of the extracts. In the evaluation for each extract, 25 mice were randomly divided into 5 groups of 5 mice/group and were given orally 500, 1,000, 2,000, and 4,000 mg/kg body weight in single dose volume of 0.2 mL of the extracts. The control group received 0.2 mL of respective vehicle of each extract (7% Tween 80 and 3% ethanol in distilled water). Then, these mice were observed continuously for 1 h, intermittently for 4 h, and for a period of 24 h for any gross behavioral changes including rigidity, sleep, mortality, and other signs of toxicity, and follow-up continued for 30 days.

### 2.6. Rodent Malaria Parasite

Drug sensitive-*Plasmodium berghei* strain ANKA (PbANKA) obtained from MR4 (Malaria Research and Reference Reagent Resource Center, EBI Resources, University Boulevard, Manassas, VA, USA) was used in this study. The cryopreservation stock was injected intraperitoneally to naïve ICR mice. Parasitemia was determined by microscopy of Giemsa stained thin blood smear. The parasite was maintained by serial passage of 1 × 10^7^ parasitized erythrocytes on a weekly basis. Percent parasitemia was calculated using the following formula:

(1) % parasitemia=Number of parasitized erythrocytesNumber of erythrocytes×100.

### 2.7. *In Vivo* Antimalarial Activity

The efficacy test of GPE, MOE, and combination with artesunate was carried out using standard protocol following Peter's 4-day suppressive test [21]. Groups of naïve ICR mice (5 mice/group) were inoculated intraperitoneally with 1 × 10^7^ parasitized erythrocytes of PbANKA. Then, GPE (500, 1,000, and 2,000 mg/kg), MOE (500, 1,000, and 2,000 mg/kg), combination of GPE (500, 1,000, and 2,000 mg/kg) and artesunate (6 mg/kg), and combination of MOE (500, 1,000, and 2,000 mg/kg) and artesunate (6 mg/kg) were administered orally to the individual groups. The control groups were also used including artesunate (6 mg/kg) treated and untreated mice as positive and negative controls, respectively. Treatment was started 3 h after infection on day 0 and then continued daily (every 24 h) as a single dose for 4 days (days 0–3). On day 4, parasitemia was estimated by microscopy of Giemsa thin blood smear, and suppression percentage was subsequently calculated using the formula given below:

(2) $$\begin{array}{c} \text{\% suppression}=\frac{\left(\text{parasitemia of untreated group}-\text{parasitemia of treated group}\right)}{\text{parasitemia of untreated group}}\times 100. \end{array}$$

### 2.8. Statistical Analysis

GraphPad Prism software was used to analyze this study. All results were expressed as mean ± standard error of mean (SEM). One-way ANOVA with Tukey* post hoc* test was used to compare several treated groups, and significant differences were considered at 95% confidence, *P* < 0.05.

## 3. Results

### 3.1. Acute Toxicity Test

The GPE and MOE administered orally in a single dose of up to 4,000 mg/kg showed no lethal effect within 24 h of observation. Gross physical and behavioral observation of these experimental mice revealed no visible signs of toxicity such as paw licking, salivation, stretching, urination, lacrimation, hair erection, and reduction in feeding activity. Generally, the mice were physically active. Additionally, no mortality occurred within the observation period of 30 days.

### 3.2. Antimalarial Activity of* G. pentaphyllum*

**Figure 1:**
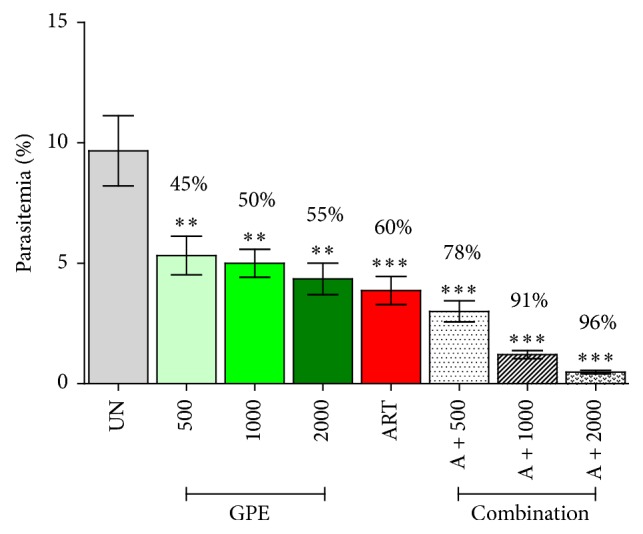
Antimalarial activity of* Gynostemma pentaphyllum*. Groups of naïve ICR mice (5 mice each) were infected intraperitoneally with 1 × 10^7^ parasitized erythrocytes of PbANKA. They were then given orally the extract (500, 1,000, and 2,000 mg/kg) either alone or in combination with artesunate (6 mg/kg) for 4 consecutive days. Untreated and artesunate treated mice were used as negative and positive controls, respectively. Parasitemia and suppression percentage were subsequently measured. ^*∗∗*^ *P* < 0.01, and ^*∗∗∗*^ *P* < 0.001, compared to untreated control. UN: untreated control; GPE:* G. pentaphyllum* extract; ART: 6 mg/kg of artesunate; A + 500: combination of 6 mg/kg of artesunate and 500 mg/kg of GPE; A + 1000: combination of 6 mg/kg of artesunate and 1000 mg/kg of GPE; A + 2000: combination of 6 mg/kg of artesunate and 2000 mg/kg of GPE. The results came from 3 independent experiments.

The effect of GPE on PbANKA infected mice, parasitemia measurement obtained from 4-day suppressive test, and percentage of suppression of parasitemia by GPE were summarized in Figure 1. Accordingly, 500, 1,000, and 2,000 mg/kg doses of GPE significantly (*P* < 0.01) inhibited parasitemia by 45, 50, and 55%, respectively, in a dose-dependent manner. The reference drug, artesunate (6 mg/kg), caused 60% suppression, which was higher than that of the extract treated groups. For combination treatment, the results indicated that GPE performed better antimalarial activity when combined with artesunate. The combination caused more significant (*P* < 0.001) suppression in parasitemia burden with 78, 91, and 96%, at 6 mg/kg of artesunate combined with 500, 1,000, and 2,000 mg/kg of GPE, respectively.

### 3.3. Antimalarial Activity of* M. oleifera*

**Figure 2:**
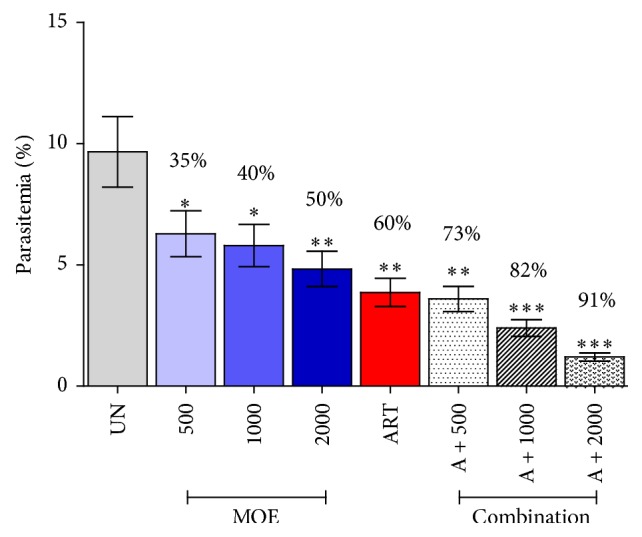
Antimalarial activity of* Moringa oleifera*. Groups of naïve ICR mice (5 mice each) were infected intraperitoneally with 1 × 10^7^ parasitized erythrocytes of PbANKA. They were then given orally the extract (500, 1,000, and 2,000 mg/kg) either alone or in combination with artesunate (6 mg/kg) for 4 consecutive days. Untreated and artesunate treated mice were used as negative and positive controls, respectively. Parasitemia and suppression percentage were subsequently measured. ^*∗*^ *P* < 0.05, ^*∗∗*^ *P* < 0.01, and ^*∗∗∗*^ *P* < 0.001, compared to untreated control. UN: untreated control; MOE:* M. oleifera* extract; ART: 6 mg/kg of artesunate; A + 500: combination of 6 mg/kg of artesunate and 500 mg/kg of MOE; A + 1000: combination of 6 mg/kg of artesunate and 1000 mg/kg of MOE; A + 2000: combination of 6 mg/kg of artesunate and 2000 mg/kg of MOE. The results came from 3 independent experiments.

As shown in Figure 2, the percentage of suppression at the doses of 500, 1,000, and 2,000 mg/kg of MOE was 35, 40, and 50%, respectively, while that of artesunate treated group was 60%. The extract caused significant (*P* < 0.05) suppression in a dose-dependent manner, compared to untreated control. In addition, combination of MOE with artesunate showed enhancement in antimalarial activity in a dose-dependent manner with significant (*P* < 0.01). The percentage of suppression at the doses of 6 mg/kg of artesunate combined with 500, 1,000, and 2,000 mg/kg of MOE was 73, 82, and 91%, respectively. Hence, antimalarial activity of MOE combined with artesunate was greater than the effect of artesunate or selected doses of the MOE when used singly in PbANKA infected mice.

## 4. Discussion

*P. berghei*-infected mice as rodent malaria model has been employed in this study. Several standard antimalarial drugs have been identified using this rodent model; therefore, it was the appropriate malarial model for the study [22–27]. Additionally,* in vivo* models are usually employed in antimalarial studies because they take into account the possible effect of prodrug and probable involvement of immune system in eradication of the parasite [28]. Moreover, the choice of 4-week-old mice for this study was used to avoid the effect of anemia in old mice and the effect of possible physiological changes associated with aging-related effects on the treatment result [29].

The present study assessed the antimalarial properties of two plant extracts,* G. pentaphyllum* and* M. oleifera*, individually and in combination with artesunate. The choice of these plants was based on the remarkable activity of quinine and the success of artemisinin [30]. However, reported cases of antimalarial resistance to these drugs made the search and development of new antimalarial drugs. The aqueous crude extracts of* G. pentaphyllum* and* M. oleifera* leaves showed high antimalarial properties with dose-dependent manner against* P. berghei* infection in mice as evidenced by the percent inhibition of parasitemia development. These extracts exhibited a dose-dependent manner. As the dose increased, likewise the antimalarial activity increased significantly. It has been described that plant extracts contain active compounds that have great potential for medicinal use, and both traditional healers and pharmaceutical drug companies make use of these plant extracts. The extracts of* G. pentaphyllum* and* M. oleifera* leaves were reported to contain different classes of active compounds such as tannins, alkaloids, quinines, saponins, flavonoids, polyphenols, terpenoids, quercetin, kaempferol, and gypenoside [31, 32]. Terpenoids, flavonoids, alkaloids, quinines, quercetin, and kaempferol were known to have antimalarial activity [33–36]. Moreover, polyphenol and flavonoids in these extracts which have antioxidant activity may also contribute to the antimalarial activity [37]. It has been described that antioxidant activity can inhibit hemozoin formation, and free heme is very toxic for the malaria parasite [38]. These active compounds which are found in the extracts may be acting singly or in synergy with other compounds to exert the antimalarial activity. In addition, quercetin, kaempferol, and gypenoside were found evidently on the ring stage of the parasite [39]. The mode of action corresponds with the nucleic acid and protein synthesis. Furthermore, anti-inflammation of quercetin, kaempferol, and gypenoside includes the nuclear transcription factor-kappa B (NF-*κ*B) inhibition, making therapeutic target for cancer treatment. The role of NF-*κ*B is also important in malaria growth and development [40]. The deciphering of antimalarial property in these extracts lays the foundation for the need to reevaluate their possible role in this transcription factor regulation for malarial control.

There are several reports on antimalarial drug combination showing their* in vitro* and* in vivo* on malaria parasites [41–45]. The aqueous crude extracts of* G. pentaphyllum* and* M. oleifera* leaves exhibited high level of antimalarial activity in* P. berghei*-infected mice when administered with artesunate. The significant decrease in parasitemia levels in response to combination treatment compared to the control, observed with the mice, was the main finding in this study. Hence, the addictive effect of these extracts with artesunate is important in the context that offers opportunities to further standardize new artemisinin-based combination therapy as possible antimalarial combination [46].

The aqueous crude extracts of* G. pentaphyllum* and* M. oleifera* leaves were well tolerated by the mice up to the dose level of 4,000 mg/kg within 24 h and up to 30 days. In acute toxicity, doses higher than 5,000 mg/kg are generally not considered as dose related, which is in accordance with the Organization for Economic Corporation and Development (OECD) Guidance Document for Acute Oral Toxicity Testing [47]. The oral median dose of these extracts were estimated to be greater than 4,000 mg/kg which was 8 times greater than the minimum effective dose of 500 mg/kg. Previous reports have shown that the median lethal dose of a test compound is 3 times more than the minimum effective dose. This compound is considered a good candidate for future studies [48]. Therefore, these aqueous crude extracts can be considered as nontoxic at acute oral administration, and the mice were safe with the different doses of these extracts administered to them.

## 5. Conclusions

This study has shown that aqueous crude extracts of* G. pentaphyllum* and* M. oleifera* leaves exhibited a significant antimalarial activity. Moreover, these extracts were safe at the highest tested dose of 4,000 mg/kg. Additionally, artesunate enhanced the antimalarial activity of these extracts. Hence,* G. pentaphyllum* and* M. oleifera* leaf extracts and their combination with artesunate show strong promise for development as antimalarial combination therapy.

## Appendix

See Figures 1 and 2.
